# Slow journalism: a letter to ethnographers

**DOI:** 10.3389/fsoc.2023.1141033

**Published:** 2023-05-04

**Authors:** Ted Conover

**Affiliations:** New York University, New York City, NY, United States

**Keywords:** ethnography, journalism, participant observation method, anonymity, privacy

At New York University I teach a graduate seminar called Ethnography for Journalists. The aim of the course, I tell my students, is not to learn how to write ethnography. Rather, I say, nobody has invested more energy thinking about humans as social beings than social scientists. What have they learned that we as journalists can borrow? I explain that it's a course in longform narrative writing and ethnographic thinking. It aims to teach students to be not an ethnographer but rather a journalist who can perceive the world like one.

The first year I taught this class, I assigned work by great anthropologists I'd read as an undergraduate, including Radcliffe-Brown, Malinowski, Geertz, and Benedict. Big mistake: I could see students' eyes glaze over as they failed to connect with the prose of titans. Soon I found a better text, Mitchell Duneier's *The Urban Ethnography Reader*, starting with its introduction, where Duneier cites “the old canard that ethnography is merely ‘slow journalism.”' Students of journalism quickly grasped what he meant when he explained that ethnography “seeks to go beyond what people might say in interviews and to reveal understandings that emerge only after countless interactions over the course of time.” That made sense, as did letting them choose their own readings from the dozens collected by Duneier.

I consider this essay to be a sort of letter to the land of bona fide ethnographers from an admirer on the outskirts—a letter of appreciation and self-explanation that might inform the space between journalism and ethnography, where I spend a lot of time. I am a working journalist, and professor of journalism, whose exposure to ethnography suggested paths toward a deeper journalism that I have pursued, and tried to teach, for many years. I am flattered when real ethnographers point to me as a fellow traveler. Yet at the same time, I know that our projects are different. Most of what follows is an accounting of the ways ethnography has empowered my journalism, though I will also speak to differences in our pursuits and the ways in which I do *not* emulate traditional ethnography. My goal here is not to critique current practice (I am not really qualified nor especially interested in doing so) but rather to describe how I do things and speak to this the space between our two traditions, which I believe contain ways to enrich each other.

The number of journalists interested in ethnography is not large. Conducting ethnographic field work takes time, and journalists (probably like most ethnographers) seem to have less of it than ever before: the rise of digital publishing and advertising have ravaged newspapers and magazines and pushed thousands of journalists out of work. Those who remain feel under constant pressure to produce not just timely content (aka, news) but clicks and page views. Then there are the things ethnographers typically do that journalists normally don't, including seeking approval from an institutional review board, masking the identities of people and places, and aiming for an academic audience rather than a general one.^1^

Still, I tell my journalism students, there is much we can learn from social scientists. I try to contextualize the various insights, lessons, and research strategies I learned from ethnographic studies of social worlds in stories I tell them from my own research. For example,

- Social worlds can be understood in terms of shared meanings, which often correlate to personal qualities the culture admires. Corrections officers, I learned at the New York State training academy, say their work is about the Care, Custody and Control of inmates. Above all, in my experience, they admire an officer's ability to *control* prisoners, especially their movements into and out of controlled spaces inside the prison.
- Social worlds can be understood in terms of status hierarchies. The railroad tramps I traveled with often shared the following ranking with me, once they learned I was new to the rails: The most admirable person on the rails is the tramp, who travels and works. Next best is the hobo, who travels but doesn't work. Least prestigious is the bum, who neither travels nor works.
- Social worlds can be understood in terms of material artifacts. Prison officers care greatly about keys (Why? Because they are instruments of control). USDA inspectors and other meat workers devote a huge amount of attention to the knives they use in a slaughterhouse (Why? Because the sharpness of a knife correlates to the effort required to cut into meat, and how much pain one feels in one's hands and arms at the end of the day).^2^
- Social worlds can be understood in terms of the meanings ascribed to certain spaces. Railroad tramps have a shared ranking of *places to ride on a train* (Protection from the wind and from the view of railroad employees are key factors). Undocumented migrants quickly acquire a keen understanding of the kinds of places they might be hassled (by police or by racists) and the kinds of places they'll be left alone.
- I talk about the tensions between etic and emic understandings. I say that the fullest understanding of a social world or subculture might come from appreciating both kinds of meaning, and the tensions between them (The general public believes that corrections officers are often brutal, for example. That explains why a corrections officer, making a casual acquaintance outside the world of prisons, might tell the new friend they work in a different occupation than they in fact do—they might say they're a contractor, in other words, or that they work in private security).
- I talk about participant-observation. I suggest that students view it as a line with two endpoints: researchers starting work on a story about a new social world begin as observers. But their goal should be to participate: to talk to people, of course, but also insofar as possible to share food with them, travel with them, work with them, or simply “hang out” with them in the spaces they inhabit. The goal is to move along the line from being an observer (who might, say, learn about football from watching TV) to being more of a participant (who might interview players in their locker room or broadcast interviews from the sidelines of a game). The more you participate the closer you come to having an insider's perspective. But of course, while you may aspire to have a participant's understanding, you never will completely succeed (and then we discuss why you won't, and how that matters).

Next semester, I will assign students the brief prolog to my recent book *Cheap Land Colorado: Off-Gridders at America's Edge* (2022). The prolog describes a moment several weeks into my research, when I'm not yet living on the prairie but I'm making progress on gaining a participant's understanding. In it, I'm the passenger in a pickup truck driven by a rural outreach worker in Colorado's expansive San Luis Valley. The very first thing that happens is that the worker explains to me how he approaches a stranger living off-grid in the remote precincts of the valley where land is very cheap. His goal is to make a connection with the prairie dweller, who well may be hostile to his presence; high on his list is putting the resident at ease, especially important because so many of them fear strangers and have firearms (“If they have a flag then they probably have a gun,” was one of his lessons). In class, I will explain how I pay lots of attention to how I present myself “in the field”—whether you're a journalist or an ethnographer, one of your key goals will be to establish rapport with people. I will say that the work of ethnographers introduced me to a research posture I have embraced to this day: *The people you're interested in know the things you want to learn; make them your teachers*.

I may also address empathy, a connection ethnographers valorize that is not always taught in journalism classrooms. Traditionally, journalism has been about objectivity (or more recently, in recognition of the biases we all bring to perception, *fairness*), about “seeing both sides.” That goal may make sense when the subject is politics, local controversies, social conflict. But when our aim is to more deeply understand a particular point of view or way of life, making an empathetic connection, “seeing through their eyes,” is a better research posture. Immersion, a term that I use as a shorthand for participant-observation, has empathy as its goal.

While ethnography has many lessons for journalists, as I've outlined above, in other respects it does not serve as a model. In the sections that follow, I'll refer mainly to my experience with *Cheap Land Colorado* in discussing issues where I see ethnography and journalism as being in tension. I'll also include thoughts on how social media and the rise of internet search have changed the landscape for this kind of research for me and other journalists.

## Social media

The people I wanted to write about in southern Colorado were mainly poor, White, and rural—but rather than tenant farmers, as in the famous account by James Agee and Walker Percy, *Let Us Now Praise Famous Men*, they are landowners: Colorado's San Luis Valley has a large number of cheap, off-grid, 5-acre lots created by sub-dividers in the 1970s, and initially sold by mail and from newspaper ads (“Buy your Colorado ranch! $30 down and $30 a month!”). They cost about $3,000 - $5,000 at creation and that has not really changed, fifty years later, such is the oversupply. Few people lived on their lots until just a few years ago, when housing in town became prohibitively expensive, solar panels got cheaper, and Colorado began to allow the cultivation of marijuana for personal use. My sister, who visited the area in connection with her work for a foundation, sent me photos of old RVs and trailers and sheds serving as residences in wide open spaces. It looked like parts of Appalachia without the trees. Donald Trump had recently been elected president and, I'd heard, was popular among many of the homesteaders. I thought that getting to know them might help me get out of my urban political silo. I began to volunteer for a local group that had begun as a rural homeless shelter; they had received money (not from my sister's employer) for a “rural outreach” initiative that aimed to help the off-gridders avoid homelessness when the weather got cold—a common fate.

I ended up buying a camper trailer and renting space for it from a family that was homeschooling five daughters on the prairie, the Grubers. After about two years of regular visits, I wrote a long article for *Harper's Magazine* about my experience. Then, feeling I was just getting started, I bought my own five acres nearby (it came with an old mobile home), and wrote a book about my four-to-five years of living part-time on the prairie.

At various points during the project I picked up *Let Us Now Praise Famous Men*, which, in terms of subject matter, intended audience, and Agee's nervousness about his position as a Harvard-educated person of privilege, seemed relevant to my project.

But Agee's three tenant farmer families seemed so different from the people I was meeting! They seemed to be largely isolated, with an experience of the world limited to their immediate surrounds. In a nutshell, they reminded me of traditional peoples from the ethnographies I had read in college, different enough from the researcher and the reader that it didn't feel like a stretch to refer to them as “subjects.” By contrast, the people I was getting to know in the San Luis Valley shared a lot of the same mass culture that I grew up with. Frank Gruber, the father of the family I rented trailer space from, has his back covered with Scooby Doo cartoon figures. He grew up a juvenile delinquent in Denver, a city where I had been an honor student; he was a fan of the Denver Broncos football team and Colorado Avalanche hockey team. His wife, Stacy, had lived mostly around Casper, Wyoming. She had worked as a waitress in a diner, on a ranch, and in a traveling carnival. When they had enough money they shopped at Walmart, just as most everyone in the valley did, or stopped by Little Caesar's Pizza. Their girls, when I first met them, were passionate about My Little Pony; more recently, they were very into cosplay and posting on TikTok. We were all products of American mass culture.

I learned some of Stacy's work history from her Facebook profile. And that points to another sea change in the lives of our “subjects”: many or most are on social media, as am I and my friends. Smart phones, with their cameras and constant connectivity, change everything. Social media not only lets me know about *them—*in the Gruber family, mom and dad and the two oldest sisters post regularly—but it lets them know about *me*. The ramifications are, of course, momentous. I can check in on them and follow their news when I'm away. They can do the same with me.

Social media also gives them the ability to praise or criticize me—not only to their world of acquaintances but, if I have “friended” them, to mine. This has made me more careful about what I post. Though I grew up in Colorado, I am today a New Yorker, a college professor, and a journalist, three identities that are not very popular on the prairie. I try not to fuel their preconceptions by sharing political comments or memes that I know would raise their eyebrows. I was pleased and relieved when the editors of *Harper's* used a photograph of Stacy Gruber for their cover photo—she loved it, and bragged on Facebook about her newfound status as cover girl. She and other family members expressed no qualms to me about the content of the article; if they had any, I think that cover photo, along with photos inside of other family members, would have blunted them.

About six months prior to publication, my book publisher created a web page for the book I had finished, now titled *Cheap Land Colorado*. I learned about the page when I saw that a person I didn't know had posted the link to it on a local Facebook group. Others I didn't know commented, some in the snide, skeptical tone one might imagine local people taking about the work of an Eastern college professor who had dared to write a book about their part of the country. But there were also comments by people I had met who defended me, urging others to reserve their criticisms until they had read the book.

As I write this, my book has been available in stores for five months. It is 100,000 words long, and I'm sure that some of those words will strike people in the valley the wrong way, and that they'll post about it to social media. That's life, and as a journalist in this millennium, I fully expect online criticism. What will likely matter more to me, though, is the feedback I receive from those whom I wrote about in the book. I gave free pre-publication copies to almost all of them, both to stanch the currents of rumor and gossip and as thanks for the hours and days they spent talking to me. Unlike many of the people in *Let Us Now Praise Famous Men*, my neighbors are literate. Some didn't graduate from high school, and I don't think I know any who graduated from a four-year college. But they're plenty smart and I'm sure I'll be hearing from them.

Do today's authors of academic ethnographies also expect feedback on social media? I expect that the answer is not so much, especially if they have masked the details of people and places to the point where people don't recognize themselves. Also, books from university presses are often quite expensive, and the delay between research and publication is usually longer than that in journalism or trade nonfiction, due in part to the time needed for peer review. My book, granted, lists for thirty dollars. But the audiobook costs less; a paperback will follow at perhaps half the price of the hardcover; and, most relevant, copies of unauthorized pdf's will likely become available on pirate sites within weeks of publication—if they aren't already! (I don't have the heart to look).

## Privacy and anonymity

Ethnographers and journalists think differently about privacy and anonymity. Ethnographers speak of protecting the people they write about by changing their names and, often, masking other identifying characteristics, such as where they live and the names of people around them. Sometimes they go so far as to create composite characters—two or more people wrapped into one. I have heard that some institutional review boards (IRBs) may *require* that professors to anonymize in order to protect vulnerable subjects. Per federal regulation, students and professors of Journalism are exempt from IRB review (One reason I have heard for this is the delays inherent in the IRB process—most topics in journalism are timely, and reporting can't wait weeks or months for approval).

For journalists, by contrast, accurate information about “sources,” including their real names, is expected practice. Exceptions can be made for people who are vulnerable or at risk (say, children, or dissidents living under a repressive regime). In the subcategory of investigative journalism, anonymity might be offered to people sharing sensitive information that could, for example, get them fired. But because accuracy is of paramount importance in journalism, granting pseudonyms or other forms of anonymity is discouraged, because not only does it release the person quoted from accountability for what they've said, it means there's no way to factcheck the journalist. A famous example of a journalist hiding falsity by depending on pseudonyms is Janice Cooke's article for *The Washington Post*, “Jimmy's World,” about a supposed eight-year-old heroin addict. It won a Pulitzer Prize but afterwards, when the deception was uncovered, it ended Cooke's journalism career. A freelance writer for the *New York Times Magazine*, Michael Finkel, sparked a firestorm when it was discovered he had combined interviews with several boys from Mali into a composite character for his article, “Is Youssouf Malé a Slave?” Rearranging chronology or making up quotations are similarly verboten.

The best magazines all do factchecking of their articles. Social scientists who write for them seem to adopt the practice: Matthew Desmond, whose stellar *Evicted* (2016) was excerpted before publication in *The New Yorker*, at the end of the book thanks his “obsessive and tireless fact-checker, [who] made this book better.”

Publishers of trade nonfiction books do more limited factchecking, and often it is focused on legal liability—but increasingly, authors of nonfiction books hire their own factcheckers, as Desmond did. Still, they are more accepting of pseudonyms than some magazines. I made up names for several corrections officers and prisoners in my book *Newjack: Guarding Sing Sing*, generally those I did not portray in a positive light; at the book's beginning is a list of all of those pseudonyms. In *Let Us Now Praise Famous Men*, James Agee and Walker Evans used pseudonyms for every person depicted, perhaps to protect their privacy (Evans used their real names with the same photographs that are archived in the Library of Congress).

I appreciate that the ethnographic tradition has embraced a sort of fuzziness around particular details of people and place, in the name of foregrounding ideas and analysis; the idea is that advances in social science are predicated on ethnographers' ability to typify and abstract. But I'm also aware of pushback in ethnography against masking, creating composite characters, and other forms of vagueness (Jerolmack and Murphy, 2019; Murphy et al., 2021). Before *Evicted*, Alice Goffman's *On the Run: Fugitive Life in an American City* (2014) was the last ethnographic book to make the leap into mainstream publishing. I was thrilled by *On The Run*, and on my invitation, Alice Goffman visited NYU Journalism to speak about it. Afterwards, the news that Goffman had embellished and possibly invented some of her subjects' encounters with law enforcement precipitated anguished discussions between me and my students. Though he didn't think Goffman “made up any data,” stated Columbia sociologist Shamus Khan, “I think there are questions about reporting things she heard as if they were things she saw (which she is hardly unique in doing – most people do this, but they definitely should not).”^3^ Most journalists, I should add, do *not* do this.

I should here note that most of my journalism takes the form of longform articles (i.e., articles longer than 3,000 words) and books. Also I tend to write narrative, which means I may follow a set of “characters” over weeks, months, or years. In many ways, this makes me a cousin to ethnographers, who might get to know a small group of people quite well. It also introduces a tension with certain practices and tenets of traditional journalism. A reporter who is in touch with a congressional staffer, for example, is likely to explicitly discuss how the information can be shared—“off the record” means it cannot be shared, while “on background” usually means it can be shared without specific attribution (“according to a congressional source …”). But with sources who are not sophisticated in the ways of journalism, a reporter needs to remind them that she is there doing a job. Ways of reminding such a source include writing down things they say when they say them, or referring back to something they said previously and clarifying some detail. Over time, people who start as strangers to us may also reveal information that is intimate or potentially sensitive. While a political reporter investigating wrongdoing might be excited to uncover dirt (“Republican candidate for U.S. Senate paid for girlfriend's abortion”), a journalist like me might need to suggest that a source be more cautious about sharing certain stories.

A final opportunity for sources to control what is written about them by longform journalists often comes during the factchecking process. Typically a factchecker will get in touch with a subject directly to verify the journalist's claims about them. Should a discrepancy arise, an editor might loop in the journalist to work out a solution.

After *Harper's* published my article in 2019, I was contacted by a reality television producer in Los Angeles. He told me he thought that the world I described in the article might be a good subject for a reality show—the genre is rich in series about off-gridders, gold miners, and wilderness explorers. I was not surprised by his interest, as I've been contacted by reality TV producers previously, as have other writers I know who write about subcultures: reality television has a huge appetite. But I was worried because sometimes the shows feel exploitative; and they require drama (i.e., emotional conflict between subjects); and it might expose people I had tried to portray sensitively to criticism on social media or even in old-fashioned media.

My first call, before I answered the producer, was to La Puente, the nonprofit I volunteered for. They didn't want any part of a reality TV deal. But they agreed with me that, if the prairie people I wrote about were interested, it should be their decision. There is, after all, money to be made by taking part in these shows. And so I started checking with the Grubers and others: did they want me to share their contact information?

Almost all of them did, though some asked to discuss the matter with me first. And in these discussions, I learned that by and large they already were well aware of the devil in the details. They watched these shows; they knew what they were about. At the very least, they wanted to “hear the man out.” As for a possible loss of privacy, they didn't seem worried. What was to be gained was perhaps money, and fame; and while they knew that getting famous could have a cost, they also knew that, for people in their circumstances, fame and money could be closely tied. Certain ones among them also saw this as a route to acquiring more social media followers, which to them was another route to fame and money.

In other words, with one or two exceptions, they were against me obscuring their identities. Most very much wanted me to use their names.

## Secretive research, secretive notes

Ethnography is also in tension with investigative journalism, and particularly with undercover reporting. As mentioned, one traditional tenet of good journalism is that journalists are working in the public interest, for the general reader. That distinguishes journalism from, say, public relations, where the goal is to burnish the reputation of a client. The journalist, by contrast, aims for fairness and objectivity.

Investigative journalists strive to uncover truths that others with vested interests might prefer stay hidden. Those interests might be corporate (e.g., big oil companies don't want to be associated with climate change) or governmental (e.g., a child protection agency might wish to avoid publicity around a missed warning or a mistaken removal). With such stories, the journalists might try to hide their true agenda from sources, in hopes of eliciting damning information. Or, in an extreme example, they might go so far as to hide their identity as a journalist in order to learn more. That is the case with undercover reporting.

Undercover reporting is controversial among journalists. Major outlets such as *The New York Times* and *The Washington Post* have policies that forbid reporters from actively misrepresenting themselves. Journalistic watchdogs who agree with this prohibition say things like, *if we know you didn't tell the truth to your sources, how do we know you're telling the truth to your readers?*

On the other hand, the potential power of undercover reporting is undeniable. Northern journalists secretly attended slave auctions in the antebellum South. Nellie Bly's feigned insanity and commitment to an insane asylum for ten days prompted hearings and reform after her report was published in 1887. Upton Sinclair's secretive visits to Chicago meatpacking houses occasioned national horror upon the publication of *The Jungle*. John Howard Griffin's travels through the Deep South in blackface, originally commissioned by *Sepia* magazine, later collected in *Black Like Me*, opened the eyes of some White people to pervasive racial discrimination.

My book *Newjack: Guarding Sing Sing* was reported secretively, as was my article about working as a USDA meat inspector for *Harper's*. While I was waiting to get hired by the USDA, I happened upon an academic study that was similarly researched: political scientist Timothy Pachirat's impressive *Every Twelve Seconds: Industrialized Slaughter and the Politics of Sight*. To research the book, Pachirat hid his identity—like me—and spent five months working in a Midwestern slaughterhouse that he doesn't name. And in the fashion of many ethnographers, he anonymizes everyone who appears in the book. As a journalist, I kept wondering, why not share the name of the slaughterhouse that employed him? Using real names lends power to accounts of a way of life. It lets a reader know this was real, I didn't make it up. To anonymize by default strikes me as potentially self-defeating: it disempowers a document. And in an age where the label of “fake news” is used to delegitimize media that speak truth to power, as a journalist, I think documentarians need to establish “facticity” wherever possible. Now that I'm in the academy myself, I appreciate that Pachirat was beholden to certain ethical obligations that I am not. The IRB presumably required Pachirat to ensure that he would minimize the harm that his research would produce for his subjects (e.g., the closure or sanctioning of the slaughterhouse)—no matter how morally repugnant the operators of the slaughterhouse may be. In contrast, journalists often celebrate if their work results in the powerful being held publicly accountable for their misdeeds.

Transparency, though, is tricky. In journalism, it has long been verboten to share a news story with a source prior to publication; the reason is that the source may ask for changes that accord with their interests but not with readers'. Fact-checkers are typically instructed not to read sentences to a source verbatim but rather to paraphrase them. However, when it comes to preparing longform articles reported through immersion, the rules can be relaxed, particularly when the story is about a vulnerable or underprivileged person. I know of two celebrated works of trade nonfiction about indigenous Americans published in recent years where the (White) writers were allowed to go over the manuscript word-for-word with their subjects, the thinking being that the subjects were particularly vulnerable, and traditional fact-checking might be less likely to unearth errors than with other kinds of work.

I'm intrigued by newer models of transparency proposed in recent years by ethnographers such as Reyes (2018), who discusses the issues involved in sharing data (e.g., interview transcripts and fieldnotes). I can see the benefits in terms of reproducibility, awareness of reflexivity, comparison over time, and, as she says, general facticity. But I have never heard a similar proposal seriously advanced for journalists or other writers of longform nonfiction. I can imagine many good reasons why a journalist would demur, including second-guessing by readers (“Why did you use that quotation instead of the other one?” “Why did you talk to Person X but not Person Y?” “Your notes reveal a complete failure to comprehend the underlying issues.”). Journalists, probably myself included, would prefer to be judged by the work itself. While we have an ethical obligation to be as judicious and fully-informed as possible, there is no presumption in journalism that one article will *build upon* previous work in an explicit way such as there is in social science/academic writing, nor that the next journalist to visit a town/organization/community should leave behind a scaffolding of primary data. I picture an inventive chef saying, *judge me by the dish, not by the recipe*. In contrast, it seems that ethnography's positioning as a social science makes it more vulnerable to demands for “data transparency” than journalism.

Some 50 years after James Agee and Walker Evans went south to report *Let Us Now Praise Famous Men*, journalist Dale Maharidge and photographer Michael Williamson located the descendants of the sharecroppers described in that classic work and wrote an update of a sort unusual in journalism. Their postscript to Agee and Evans' work, *And Their Children After Them*, won the Pulitzer Prize for General Nonfiction in 1990. Maharidge then became a tenured professor at the Columbia Graduate School of Journalism. I've always been impressed by their achievement. But in journalism, such explicit nods to the greats are rare.

## Audience

Mainstream journalists aim to reach a wide audience of general readers. Those working in the digital sphere may measure impact by page views and clicks; ad sales; and subscriptions. The coin of the realm is breaking news, what's happening now. Longform narrative writing, or literary nonfiction, as it's sometimes called, is practiced by people who may as readily call themselves writers as journalists. We are interested in telling in-depth stories that relate to what is happening in global culture but we are a few degrees separated from news of the day. Our stories might be historical or biographical; they might be investigative and political; they may speak to cultural trends, such as populism or #MeToo or the reparations movement. I think it is accurate to say longform writers have aspirations of creating work that lasts beyond the current news cycle. And many hope also to create writing that is graceful and beautiful—not simply a “just the facts” account but stylistically appealing. Some of us have connections to universities but most do not.

Ethnographers, by contrast, exist mainly in universities. And while sometimes their work may find a readership beyond the academy (Margaret Mead comes to mind, and Claude Levi-Strauss), most write for other social scientists. Data collection and analysis are prioritized. The use of specialized language is characteristic of much ethnographic writing, which is often in the service of building theories, comparing across cases, or identifying mechanisms—things that are usually not central to journalists and other nonfiction writers.

By contrast, I teach my students to tell stories—stories that relate to a bigger idea or topic, be it undocumented immigration, the incarceration boom, or the rise of Donald Trump—but stories just the same, because a main goal is to engage the general reader in a piece of longform writing. To get them in the spirit, I suggest an exercise that may strike an ethnographer as vampirish: read your chosen excerpt from Duneier's *The Urban Ethnography Reader* closely, and consider reading the longer work it was taken from. Then answer this question: if you had the access to people that X ethnographer did, can you imagine reporting out a more journalistic piece of writing—one more accessible to a general reader? Is there a character or two they might get to know? Is there a scene or two they could imagine including in a narrative account? Are there ways you could spend time with these people and *report for story?* In the next class, I try leading a further discussion into what might be lost in a narrative account, which ideas might fall by the wayside, and the way that the costs of simplification must be weighed against the benefits.

## A note on “subjects”

In a time when even people in the humblest of circumstances use smart phones and publish on social media, when questions of privilege and representation are high on the public agenda and certain traditions of journalistic and ethnographic practice are being reevaluated, I'm wondering: Should ethnographers still use the term “informant?” I imagine, I hope, that it is going away, because of its hint of snitching for cops and its suggestion that “the significance of this person is that they tell me things.” A cognate term in journalism is “sources.” It again connotes “the person who gave me my information” but to my ear it is similarly othering. It suggests an extractive research posture. Another term, “subjects,” feels more benign but is still troubling, because it says, *I am the studier, you are the studied*.

Matt Little, the Rural Outreach Initiative worker whom I shadowed when I first got involved with La Puente, and later emulated, had a similar problem with the terminology of his workplace. La Puente is a social service organization that typically refers to the people it helps as “clients” (Clients who stay or eat at the shelter might also be called “guests”). Matt, who lived out on the prairie in a humble trailer like the people he tried to assist, actively resisted describing his work as “serving clients.” “It's not a restaurant,” he explained. Rather, he said, he felt like he was helping out his neighbors. More often, he would refer to helping out his “people” (“One of my people could use some firewood”). He also didn't like the positionality of the “serving clients” approach, because to him it connoted something close to charity. Rather, he liked to think of the help he rendered as mutual aid, neighbors helping neighbors.

Hanging around Matt and La Puente, I was fascinated by a value they seemed to share: both were against “judging.” You could never know why someone had ended up in the tough situation they had—maybe they were careless or lazy or codependent but really it didn't matter. Circumstances had conspired against them. You couldn't know and you shouldn't judge. What mattered was, they needed help. Even more impressive was when the people receiving help were angry, or dismissive, or even hateful of you or others: Matt and the La Puente workers didn't shout back. Privately, you might occasionally hear a cavil or reservation about a client: *She's too ask-y*, I heard one manager say about a woman who did, indeed, ask for help month after month after month. But seldom did they condemn or write off anyone, and never did I witness them doing that publicly. They didn't judge.

But what about me? Wasn't it my job as an educated person to exercise critical intelligence—to analyze and even theorize?

I think my peers and I would say here that, more than analysis, we depend on the power of story to carry meaning. Like ethnographers, we pick people to write about who stand for something larger. It might be unofficial immigration, the self-isolation of the rich, the boom in incarceration, the anger of working-class Whites. Our research, then, is in service to telling a story, and the story has a point of view. Meaning is conveyed by tone and by what we include or ignore; and in my case, by the situations I put myself in and then recount. The idea is always there but particularity, and true detail—what actually happened—take center stage.

## Ethics statement

The author's works discussed in this article were researched in accordance with professional standards and institutional requirements. All identifying information relating to the research participants has been previously published, with consent from the individuals involved.

## Author contributions

The author confirms being the sole contributor of this work and has approved it for publication.

## Footnotes

^1^A work of literary journalism that draws more heavily on the work of ethnographers than any I know (and cites them all in extensive source notes) is Anne Fadiman's The Spirit Catches You and You Fall Down: A Hmong Child, Her American Doctors, and the Collision of Two Cultures (1997).

^2^To research an article for *Harper's Magazine*, I spent two months working as a USDA inspector at a Cargill Meat Solutions plant in Nebraska.

^3^Some reports suggest that Goffman *did* make up data, as here: https://www.phillymag.com/news/2015/06/11/alice-goffman-book-philadelphia-on-the-run-criticism/.
